# Multidimensional experience of pain in adults with cirrhosis: A qualitative descriptive study

**DOI:** 10.1080/24740527.2025.2573273

**Published:** 2025-12-15

**Authors:** Franklin F. F. Gorospe, David Wong, Elizabeth Lee, Lissi Hansen, Martine Puts, Craig M. Dale

**Affiliations:** a Daphne Cockwell School of Nursing, Toronto Metropolitan University, Toronto, Ontario, Canada; b Lawrence Bloomberg Faculty of Nursing, University of Toronto, Toronto, Ontario, Canada; c Perioperative Services, Toronto General Hospital, University Health Network, Toronto, Ontario, Canada; d Toronto Centre for Liver Disease, Toronto General Hospital, University Health Network, Toronto, Ontario, Canada; e School of Nursing, Oregon Health & Science University, Portland, Oregon, USA; f Tory Trauma Program, Sunnybrook Health Sciences Centre, Toronto, Ontario, Canada; g Centre for the Study of Pain, University of Toronto, Toronto, Ontario, Canada

**Keywords:** Pain, qualitative study, liver disease, biopsychosocial, multidimensional, cirrhosis

## Abstract

The incidence of liver disease is projected to increase significantly by 2030, affecting 1.5 billion of the global population and approximately 3 million in Canada. With various etiologies, the progression of liver disease to cirrhosis varies from months, years, or decades. Experiencing pain can severely debilitate a person living with this condition. However, there is limited qualitative evidence to understand the subjective pain experience of individuals with cirrhosis. Understanding this experience with cirrhosis is crucial for providing comprehensive, person-centered care. The aim of this study was to qualitatively explore the multidimensional experience of pain for persons with cirrhosis. A qualitative descriptive approach was used, recruiting participants from a hepatology clinic in Toronto in 2021. Participants completed a Brief Pain Inventory Short Form questionnaire and a semistructured interview. Data analysis was guided by Hsieh and Shannon’s stepwise directed content analysis. Fifteen interviews were conducted with adults (mean age: 54 years, 53% men, 47% women) with cirrhosis, most unable to work, with approximately half married/partnered. Participants reported diverse physical symptoms including visceral and musculoskeletal pain, often described as a constant experience. Psychologically, pain contributed to significant fatigue and emotional distress, affecting self-care and daily activities. Socioculturally, pain disrupted social interactions and financial stability, intensifying reliance on support systems. Participants reported limited effectiveness of pharmacological interventions, reliance on mindfulness and rest, and frustration with unmet pain management needs. These interrelated themes collectively impaired quality of life and independence. Exploring the multidimensionality of pain for persons with cirrhosis provides valuable insights to address the gaps in current pain management strategies.

## RÉSUMÉ

L’incidence des maladies du foie devrait augmenter de façon marquée d’ici 2030, touchant près de 1,5 milliard de personnes dans le monde, dont 3 millions au Canada. La progression de la maladie hépatique vers la cirrhose, dont les étiologies sont multiples, peut s’étendre sur des mois, des années ou même des décennies. La douleur associée à la cirrhose peut être fortement invalidante. Pourtant, on dispose de peu de données qualitatives permettant de comprendre l’expérience subjective de la douleur chez les personnes atteintes de cirrhose. Une compréhension approfondie de cette expérience est essentielle pour offrir des soins intégrés et véritablement centrés sur la personne. Cette étude avait pour objectif d’explorer qualitativement l’expérience multidimensionnelle de la douleur chez les personnes atteintes de cirrhose. Une approche descriptive qualitative a été utilisée. Les participants ont été recrutés dans une clinique d’hépatologie à Toronto en 2021. Ils ont complété le questionnaire *Brief Pain Inventory* et ont participé à un entretien semi-structuré. L’analyse des données s’est appuyée sur l’analyse de contenu dirigée en étapes, selon Hsieh et Shannon. Quinze entretiens ont été menés auprès d’adultes atteints de cirrhose (âge moyen : 54 ans; 53 % d’hommes, 47 % de femmes), dont la majorité étaient inaptes au travail et dont environ la moitié étaient mariés ou vivaient en couple. Les participants ont rapporté divers symptômes physiques, incluant des douleurs viscérales et musculosquelettiques, souvent décrites comme une expérience continue. Sur le plan psychologique, la douleur contribuait à une fatigue importante et à une détresse émotionnelle, affectant les soins personnels et les activités quotidiennes. Sur le plan socioculturel, elle perturbait les interactions sociales et la stabilité financière, accentuant la dépendance aux réseaux de soutien. Les participants ont souligné le soulagement limité offert par les interventions pharmacologiques, le recours à la pleine conscience et au repos, ainsi que la frustration liée à des besoins non satisfaits en matière de prise en charge de la douleur. Ensemble, ces thèmes interdépendants témoignent d’une altération marquée de la qualité de vie et de l’autonomie. L’exploration du caractère multidimensionnel de la douleur chez les personnes atteintes de cirrhose apporte un éclairage essentiel pour combler les lacunes actuelles dans la prise en charge de la douleur.

## Introduction

Cirrhosis, the end stage of liver disease, involves severe scarring and impaired liver function, resulting from genetic, viral, or chemical injuries to the liver’s functional tissues.^1,2^ Disease progression diminishes the liver’s ability to perform metabolic functions, metabolize toxins, and synthesize blood proteins, leading to life-threatening complications.^3–5^ The timeline for progression to cirrhosis varies from months to decades, depending on the etiology.^1,2^ Liver disease, encompassing conditions that progress to cirrhosis, is a growing global health burden, affecting an estimated 1.5 billion persons worldwide in 2017^6,7^ and approximately 3 million Canadians.^8^ From 2016 to 2030, projections indicate a significant rise in liver disease prevalence across all countries.^9^ In Canada, liver disease ranks among the top ten leading causes of death, highlighting its public health significance.^10^ The impact of cirrhosis on quality of life is profound, because it often leads to debilitating symptoms that interfere with daily activities, employment, and social relationships, significantly reducing independence and well-being.^3,11^

Pain among adults with cirrhosis is highly prevalent. Pain is defined as “an unpleasant sensory and emotional experience associated with, or resembling that associated with, actual or potential tissue damage” by the International Association for the Study of Pain.^12(p1977)^ Reports of pain among surveyed cohorts range from 79% to 100%.^8,11,13^ Notably, reported pain intensity levels range from low to severe in published studies, particularly among patients with cirrhosis.^8,11,13^ Despite the frequency and intensity of reported pain, Gorospe et al.’s^13^ scoping review showed that the documentation of pain intensity by researchers was uncommon; merely 14% of the studies included such data. The clinical manifestations of pain associated with cirrhosis encompass a spectrum of symptoms including abdominal, back, and musculoskeletal pain.^11,13,14^ However, little is known about how patients with cirrhosis experience pain from their own perspective.^13,15^ Given the highly personal nature of pain,^16^ exploration of patient experiences is a requisite first step in improving and managing pain.

The experience of living with pain is a complex phenomenon impacting multiple domains.^17^ The presence of pain can interfere with the person’s capacity to engage and participate in activities of daily living.^8,13,18^ For instance, pain can interfere with the person’s sleep and ability to ambulate. Furthermore, pain can impede paid employment and the maintenance of close relationships.^19–26^ Though pain interference in multiple domains may be explained by the complications arising from the physiological changes in cirrhosis, how the person experiences these changes is critical to understand their needs beyond their physiological status.

Available qualitative studies focus on broad concepts such as living with liver disease,^27–29^ signs and symptoms,^30^ and health care service gaps.^31^ Understanding the person’s sensory and emotional experience may be limited by unidimensional pain appraisal tools, such as numerical rating scales,^32^ which fail to consider other modifiable pain domains,^33,34^ Similarly, quantitative studies limit the person’s capacity to voice their experience living with pain. There is an intricate relationship between the physical, psychological, and social dimensions of a person, making the person’s experience a critical source to understand the multidimensionality of pain.^17,19,35^ Therefore, the aim of this study was to qualitatively explore the multidimensional experience of pain for persons with cirrhosis.

## Materials and methods

### Design

We employed a qualitative descriptive approach^36−38^ to study the multidimensional pain experience of participants with cirrhosis. The qualitative descriptive approach provides a straightforward description of a phenomenon, staying close to the data and the surface of participants’ experiences, making it suitable for exploring understudied perspectives such as pain in cirrhosis. Moreover, this approach is considered useful in its compatibility with existing conceptual frameworks for analysis.^39,40^

### Sampling and recruitment

Convenience sampling occurred between May and September 2021 among patients completing a pain survey from the ambulatory hepatology clinic at Toronto General Hospital, Canada. Recruitment involved distributing information flyers and sending invitational letters. Patients who expressed interest to their health care provider were connected either in person or virtually to a research team member. Participants were required to be 18 years or older, have a diagnosis of cirrhosis as established by a known Model for End-Stage Liver Disease and serum sodium concentration (MELD-Na) score to calculate the severity of liver disease,^41–45^ and be able to communicate verbally in English. Exclusion criteria included a history of liver organ transplantation or a suspected or confirmed cancer diagnosis.

### Consent and ethics

Eligible participants provided written informed consent prior to participation in the interview. The Research Ethics Boards of the University Health Network (No. 20-6326.1) and University of Toronto (No. 40994) approved this study.

### Guiding study framework

**Figure 1.**
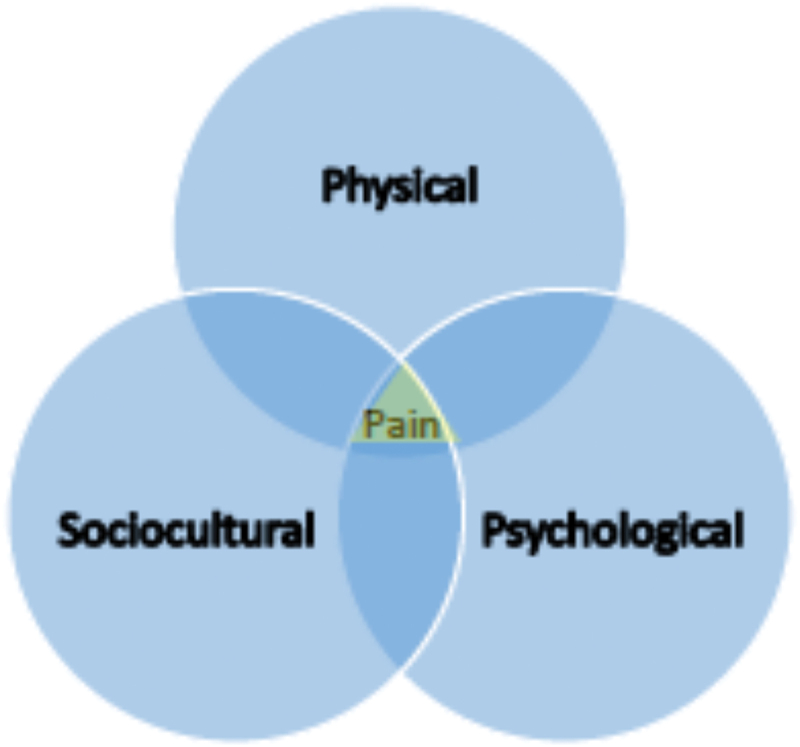
Biopsychosocial model of pain.

Turk and Gatchel’s^21^ biopsychosocial model offers a framework that views pain as a multidimensional experience of physical, psychological, and social domains (Figure 1).^13,21^ The physical (bio-)^21^ domain encompasses nociception, where an event such as illness triggers pain receptors. These generate neural signals that are interpreted by the person who actively assigns meaning to the event. In the psychological (-psycho-)^21^ domain, cognitive and emotional elements, such as coping strategies and feelings like frustration or distress, shape the pain experience. Furthermore, feelings of fatigue can also influence a person’s experience with pain.^21^ The sociocultural (-social)^21^ domain takes into account the diverse social and cultural factors, including external influences such as activity (e.g., work) and social support (e.g., marriage, family, friends). Though categorized separately, these domains are in constant interaction with each other, suggesting that the complexity of pain is inherent in the dynamic process of a person’s unique experience.^13^

### Data collection

A sociodemographic survey was conducted to collect data on participants’ age, sex, education, employment, income, marital status, and frequency of social interactions, providing context for their pain experiences (Table 1). The semistructured interviews were video recorded with the camera turned off through Microsoft Teams due to COVID-19 social distancing restrictions during the data collection period. The semistructured format allowed the interviewers to follow a guide with core questions aligned with the Brief Pain Inventory–Short Form (BPI-SF) while enabling flexibility to probe or explore participants’ responses for deeper insights into their pain experiences.^22–25,28,47,48^ The interview guide was closely aligned with the BPI-SF focused on the multidimensional experience of pain (see Supplemental Appendix A). The interview guide was tested during the initial three interviews to assess the timing, wording, and sequencing of questions. Participant feedback was considered; however, no revisions were necessary. Considering that the health status of the participants varied, the interviewer (F.G.) employed time for rest as needed. The interviewer took notes during and after the interviews to supplement the transcripts, providing additional contextual information for data collection.^38,49^ The recordings were transcribed verbatim and checked for accuracy by two team members (F.G., C.D.). Sample size was determined by data saturation, achieved when no new themes or codes emerged from interviews, occurring after 15 participants.^37,38^

**Table 1.** Participant demographic characteristics and clinical features (*n* = 15).

Characteristics	n (%)
Age	
20–39	2 (14)
30–39	1 (7)
40–49	3 (20)
50–59	4 (27)
60–69	5 (32)
70–79	1 (7)
Sex	
Male	8 (53)
Female	7 (47)
Education	
High school or equivalent	2 (13)
Some college or university	4 (27)
University degree	7 (47)
Master’s degree	2 (13)
Employment	
Not able to work	9 (60)
Retired	3 (20)
Working	3 (20)
Yearly income	
<$20,000	6 (40)
$20,000–$49,999	2 (13)
$50,000–$79,999	4 (27)
>$80,000	3 (20)
MELD-Na score^a^	
9–16	8 (53)
17–20	4 (27)
21–22	2 (13)
23–31	1 (7)
Primary liver disease	
Viral hepatitis	3 (20)
Alcohol related	5 (32)
MASLD	1 (7)
Metabolic dysfunction–associated steatohepatitis	1 (7)
Primary sclerosing cholangitis	3 (20)
Autoimmune	1 (7)
Unknown cause	1 (7)
Physical symptoms	
Ascites	14 (93)
Edema	13 (87)
Muscle wasting	13 (87)
Psychological symptoms	
Depression	3 (20)
Anxiety	4 (27)
Sociocultural features	
Marital status	
Single	6 (40)
Married or have a significant other	7 (47)
Separated	2 (13)
Socialize with family or friends	
Infrequent	3 (20)
Once a week	2 (13)
Twice a week	2 (13)
Four times a week	1 (7)
Five times a week	2 (13)
Seven times a week	5 (32)

^a^The MELD-Na score is used to predict the risk of mortality in patients with end-stage liver disease. Higher scores are associated with increased risk of death within 90 days.^46^

**MELD-Na <17**: <2% relatively lower risk of death.

**MELD-Na 17–20**: 3–4% low risk of death.

**MELD-Na 21–22**: 7–10% moderate of death.

**MELD-Na 23–31**: 14–32% high of death.

### Process for analysis

The research team employed the BPI-SF and the three domains of Turk and Gatchel’s^21^ biopsychosocial model to guide the directed content analysis: (1) physical, (2) psychological, and (3) sociocultural experiences with pain. We used directed content analysis to analyze the data, because this method enables a structured, theory-driven approach while allowing for a descriptive and authentic representation of the participants’ experiences. This method was selected for its ability to provide a conceptual framework that supports the development of priori codes to organize and analyze the data. The deductive approach used predetermined themes (physical, psychological, and sociocultural) based on the biopsychosocial model, while allowing for development and refinement of subthemes from the data to reflect participants’ experiences.^36^ Therefore, we employed Hsieh and Shannon’s^36^ stepwise approach where two members of the research team (F.G., C.D.) read the transcripts and independently identified key content. During the iterative data analysis process, codes were developed (e.g., limited treatment options) and refined. Some codes were collapsed to better reflect the emerging subthemes and to streamline the analysis. For instance, various physical pain symptoms were combined under a broader “visceral pain” subtheme. Code descriptions were developed and refined through an iterative process. Intercoder agreement was established through regular meetings and discussions, first between F.G. and C.D. (including resolution of discrepancies via consensus, review of analytical findings, refinement of interpretations, and discussions of emerging subthemes to ensure credibility) and then among the entire research team. The larger research team reexamined the identified codes to assess accuracy and relevance. Through this iterative process, the team expanded insights into the subthemes and aimed to represent the concept of interest as naturally as possible.^37–40^ Final consensus on content alignment with Turk and Gatchel’s^21^ biopsychosocial model of pain and BPI-SF’s pain interference domains^22−25,28,47,48^ was reached through discussion with the larger research team.

To facilitate the quality of reporting for this qualitative study, we utilized the Consolidated criteria for Reporting Qualitative research checklist (see Supplemental Appendix B). NVivo 12 software was used to manage the transcribed interview data and facilitate the coding.^50^

### Trustworthiness and rigour

To ensure the trustworthiness of the study, we employed strategies including triangulation, maintaining an audit trail, and addressing researcher bias through reflexivity.^36–39^ Triangulation involved using multiple data sources (e.g., video recordings, transcripts, and interviewer notes). An audit trail documented the research process and decisions. Reflexivity was practiced by F.G. and C.D. through reflective journals and team discussions to allow for awareness of thought processes, ensuring credibility and confirmability of findings.

## Results

### Participants

There were 17 eligible participants invited to the study and 15 participants completed the interview, ranging between 28 and 60 min in length. Two opted not to participate due to personal reasons. There were eight (53%) men and seven (47%) women, and the mean age was 54 years (see Table 1 for all characteristics). Many of the participants were not able to work. Approximately half were married/partnered, and 5 were socializing with family or friends seven times a week. In terms of diagnosis, 5 participants had alcohol-related liver disease, 3 participants had viral hepatitis, and 3 participants had primary sclerosing cholangitis. In terms of symptoms of cirrhosis 14 participants reported ascites, 13 edema, and 13 muscle wasting. The most frequent reported MELD-Na scores were those between 9 and 16, within the possible range of 6 to 40, where the MELD-Na score quantifies liver disease severity and predicts mortality risk, with higher scores indicating worse prognosis.^42,43^

### Qualitative results

**Figure 2.**
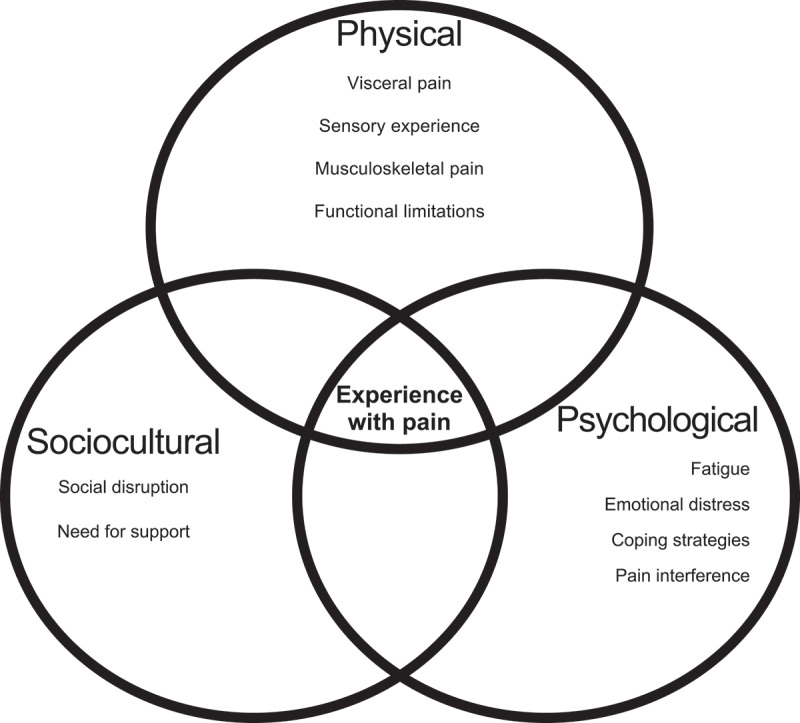
Multidimensional experience of living with pain.

The physical experience with pain theme comprises four subthemes: visceral pain, sensory experiences, musculoskeletal pain, and functional limitations. The psychological experience with pain theme is made up of four subthemes: fatigue, emotional distress, coping strategies, and pain interference with mood, sleep, and enjoyment of life. The sociocultural experience with pain theme consists of social disruption and need for support (see Figure 2). Table 2 outlines details of themes and subthemes.

**Table 2.** Themes and subthemes related to participants’ experiences with pain.

Theme	Subtheme	Participant quotes
Physical (bio-) experience with pain	Visceral pain	“Uh, so, uh, generally in two spots in my abdomen. One is kind of on my left upper left side of my abdomen. And the other one is, uh, in the upper, uh, right, uh, flank area, like, uh, the back of my, uh, on my back, almost on my, uh, rib cage.” (48-year-old, male, MELD-Na score of 9, primary sclerosing cholangitis)
		“My stomach’s pretty big, so it has an overall effect of putting pressure on just about every part of internal organ that I have and, of course, my back. It’s pushing on everything. I’m not gonna be walking anywhere at that point when it’s a 10.” (64-year-old, male, MELD-Na score of 19, viral hepatitis)
		“Yeah, everything starts to hurt ’cuz I bloat. I don’t know why I keep blowing up. I don’t understand it.” (56-year-old, female, MELD-Na score of 18, viral hepatitis)
	Sensory experience	“Probably the last year I’ve had the pain in the abdominal. I’ve had the disease for like 7 years and it was kind of quiet, like I didn’t even really know I had it, but progression when the fluid started to build up more. Um, then the pain started to come. I don’t know how you can get any higher in pain than I dealt with there, because, as I say, I’ve dealt with pain all my life so anybody that says this won’t hurt you or it’s just a little minor thing. Trust me, it is not, uh, um, it’s not okay now that it’s unbearable.” (67-year-old, male, MELD-Na score of 22, MASLD, formerly known as fatty liver or nonalcoholic fatty liver disease)
		“Because of the constant aching pain and the frustration that it caused that I was not able to complete tasks. I have no other term but frustration and a bit of anger.” (53-year-old, female, MELD-Na score of 9, unknown cause of cirrhosis)
		“I would say on a scale of 1 to 10, constantly I would say probably 6 to a 7. Ah, I would say it would go up to a 10.” (58-year-old, male, MELD-Na score of 20, metabolic dysfunction–associated steatohepatitis, formerly known as nonalcoholic steatohepatitis)
		“Yeah, so my itching is really bad and I would say that’s the worst symptom I have. I get pretty bad scars on my legs and feet and I also get super itchy on my palms of my hands and my, like, upper arm area. And then most recently I’ve been getting itchy right where my carotid artery is in my neck. And my nose itches occasionally. So those are like my itching spots. But really, the legs and the feet and the hands are the worst of it.” (21-year-old, female, MELD-Na score of 12, primary sclerosing cholangitis)
	Musculoskeletal pain	“Uh, you know. Coincidentally, I don’t know if it’s a side effect or not, but I’ve developed a back sprain. I’ve gotten degenerated disc problems in my back and I don’t know if that’s all part of the liver or not, but it’s—it bothers me. It catches me. It’s just started here about a week or so ago where and it’s, it’s, uh, it’s painful. It’ll catch me, too, and I had seen a chiropractor before for that, but I’m contemplating maybe going back and and seeing him as well so when the stomach and scrotum get big, it makes the back pain worst.” (77-year-old, male, MELD-Na score of 14, alcohol-related liver disease)
		“The abdomen is a constant discomforting pain at this point. It has gotten, uh, it has been a lot worse. At the joint is a constant, uh, it’s as if it’s, like, I’m having an arthritis flare right now, so that—that hurts more in—in back and neck is a constant as well. Since, uh, since the, uh, since the abdomen started swelling up.” (51-year-old, male, MELD-Na score of 16, alcohol-related liver disease)
	Functional limitations	“I used to love shopping, doing the grocery shopping. I can’t do that anymore because of the pain.” (64-year-old, female, MELD-Na score of 18, viral hepatitis)
		“Um, I won’t go out and/or I’ll make sure that it’s a small group of friends I’m with. If I’m in pain so that if I have to like lay down and put my feet up, they, like, they don’t care. They’re my best friends. Um, but usually I just won’t go if I’m in pain. I’d rather stay home.“ (31-year-old, female, MELD-Na score of 16, primary sclerosing cholangitis)
		“If it’s like today, it’s not bad, but at 6 it’s fine. You know it’s when it’s like at 10 or whatever, like, say, when it gets like really, really bad. I ain’t gonna be walking. I’ll be here. I’ll be sitting in the backyard getting some sun and that’s it.” (43-year-old, female, MELD-Na score of 27, alcohol-related liver disease)
		“I got walking pain. I don’t walk.” (63-year-old, male, MELD-Na score of 9, alcohol-related liver disease)
		“I can’t do any of it. I can’t work outside the yard. I can’t even help my wife put dishes away without the extreme pain after dinner and—and God bless her. She’s—she’s gotta carry the load on all that stuff because I—I just physically can’t do any of the chores around the house that I used to do [crying]. And I used to help clean the house. I used to. Well, I just can’t do any of that now, so it’s—it’s come to a full stop [crying].” (77-year-old, male, MELD-Na score of 14, alcohol-related liver disease)
		“And if you’re experiencing pain in your stomach and in your back, forget it. You’re not gonna, you can’t work, you just can’t not like an 8-hour shift.” (64-year-old male, MELD-Na score of 19, viral hepatitis)
Psychological (-psycho-) experience with pain	Fatigue	“I don’t get enough energy. So when you’re in pain, you just relax somehow or it can get really bad.” (64-year-old, female, MELD-Na score of 18, viral hepatitis)
		“You might walk through two minutes in a mall and you’re tired, whereas you could have walked for hours before, so it depends. It’s you not able to. But the only thing I can see is my elbows are okay. And, uh, my shoulders are okay, but, uh, every other part of my body it’s giving me pain. In one of those areas, the feet, the stomach—the stomach is the most extreme because it stretches like a balloon being blown up and when it gets that balloon gets completely going up.” (67-year-old male, MELD-Na score of 22, MASLD)
	Emotional distress	“So I feel like I’ve never really gotten a very concrete answer as to why, uh, you know, why, you know, I’m getting pain there. You know I’ve had a couple of, you know, different doctors kind of offer some kind of educated guesses as to what those reasons are. But, I mean, that’s one thing that you know would be nice if could I actually know, you know, why I get the pain every, you know, uh, in that side. Like the one on the—new one on the right side, you know, I’m really not sure, you know, what the genesis of that pain is.” (48-year-old, male, MELD-Na score of 9, primary sclerosing cholangitis)
		“Um, not knowing the—not knowing the cause of the pain affects you physically, emotionally, mentally because you’re unaware of what’s causing it and you don’t have the resources to speak to anyone, uh, to, to understand what is happening. That—that’s about it. That’s the only part that really affected me more than the pain, because the pain I know if I take medication, the pain may go away, but not knowing what’s causing the pain is worse than the pain itself.” (53-year-old, female, MELD-Na score of 9, unknown cause of cirrhosis)
		“Uh, it might occur maybe once a day and last for a couple hours, but it will be acute and intermittent, so it’ll come and go within that hour. Um, and it’s quite intense when it happens, almost like a migraine, but it’s not.” (65-year-old, male, MELD-Na score of 22, autoimmune hepatitis)
	Coping strategies	“Just the thought of. I just didn’t, I just [crying]—when I get talking about what I put my family through I get over emotional, but I’m fine. I just wish all of this hurting goes away. I [crying] can’t even help my wife. I’m sorry. Its just I get so down over this. It doesn’t feel right that my wife does everything and I sit around doing nothing.” (77-year-old male, MELD-Na score of 14, alcohol-related liver disease)
		“Well, it’s just tough to be happy when you’re in pain. So, um, yeah, I’ll just be less inclined to be social. More of an introvert. Um, you know, if I’m dealing with pain in my leg. Let’s at my capacity. I don’t really feel like I can do much else.” (31-year-old, female, MELD-Na score of 16, primary sclerosing cholangitis)
		“I have Tylenol, and is that healthy? Well, it does it. It helps me sleep. I try to take it at nighttime because I think it kills some of the pain, but it doesn’t relieve it totally ’cause it’ll still wake me up, but it allows me because of the poor sleeping habits I’ve gotten into or lack of sleep, then it allows me to close my eyes and sleep for a bit, so the Tylenol is the only painkiller I’m taking.” (77-year-old, male, MELD-Na score of 14, alcohol-related liver disease)
		“Like you get a headache. You take it on, it goes away, but if you take a Tylenol the headache is still there.” (67-year-old, male, MELD-Na score of 22, MASLD)
		“Cold showers used to help me a lot and I would shower like twice or three times a day last year. And now that doesn’t help me as much. Um, so I find that in the super hot weather I get really itchy, which is why I like the cold helps. Um, but then I also am really itchy in the winter, too, so maybe it’s, like, just the dry skin. And I also can tell that I’m more jaundiced sometimes when my itches is bad. So, like, sometimes the symptoms that go together. Right now I just do cold showers and lotion, and distraction really helps.” (21-year-old, female, MELD-Na score of 12, primary sclerosing cholangitis)
		“We had a nice barbecue yesterday and everything, you know, so, you know, I was experiencing pain, but I was outside enjoying myself at the same time. Like I say, it—it’s not an easy thing to do, but once you you’ve experienced pain for as many years as I have—I’ve been experiencing pain for a long, long time, and like I say, after a while you have to get used to it.” (64-year-old, male, MELD-Na score of 19, viral hepatitis)
		“Like I said, pain is, uh, that, that—that’s a four-letter word and I’d say more irritant the pain. And I’m just saying it to be honest with you. For me it’s a, it’s a pain that I can’t go running. It’s a pain that I can’t physically do things that I was able to do.” (63-year-old, male, MELD-Na score of 9, alcohol-related liver disease)
	Pain interference	“It has affected it quite a bit. I’ve become very moody sometimes. You know my household members don’t even wanna be around me. Yeah, because I’m I’m basically what they call as very grouchy and moody, and that’s probably due to the pain thing.” (58-year-old, male, MELD-Na score of 20, metabolic dysfunction–associated steatohepatitis)
		“We’re feeling good today, and, and that might last for two hours and then be done, but you just go with the goodness of not having pain. And if, if—and if you’re feeling bad when it hurts, yeah, it, it’s—it’s totally horrible, it, um, it affects you psychologically as well as physically.” (49-year-old, female, MELD-Na score of 15, alcohol-related liver disease)
		“A lot of pain has affected my sleep. Like I went four nights in a row with no sleep because my stomach hurts. I was getting so uptight. I tried everything to sleep and everything. It wasn’t happening, so …” (56-year-old, female, MELD-Na score of 18, viral hepatitis)
		“Um, recently, I’ve been waking up in the middle of the night in pain, not being able to go back to sleep, and I’ve never really experienced it. Waking me up. So that’s sort of a new thing that’s come up. Um, and I usually have it the other way around, where I can’t fall asleep because of the pain.” (21-year-old, female, MELD-Na score of 12, primary sclerosing cholangitis)
		“Now sure, I mean, from the basics of housekeeping, cleaning. Washing dishes and that sort of thing that I haven’t done for a long time to more life enjoyable things like meeting with friends or gardening, uh. Even if it’s just potted plants rather than the garden itself, uh, I—I’m unable to find pleasure in them. And I live in front of a huge park and just sitting out and looking at the park is not enjoyable anymore.” (51-year-old, male, MELD-Na score of 16, alcohol-related liver disease)
		“Well, not being able to do the things that you are accustomed to doing, it’s frustrating. And so it’s management of what you can actually do versus what you wish to do. I’m retired and I have interests in projects and so on that I enjoy doing and had great plans for achieving many of them. I just can’t do them.” (65-year-old, male, MELD-Na score of 22, autoimmune hepatitis)
Sociocultural (-social-) experience with pain	Social disruption	“Last year when I started feeling weak, uh, but before all this I swam. I used to go to a gym three times a week. I was in a boot camp. I used to camp to garden. I’m sorry, I was quite active. I was going to school and working and quite active and right now it’s nothing. I don’t do any any of that. I met with friends all the time and went to places with them. I had them over and none of that is happening. None of that has happened for quite some time.” (51-year-old, male, MELD-Na score of 16, alcohol-related liver disease)
		“Yep, uh, so the pain comes up mostly when I, uh—that’s swollen feet or swollen legs. It’s uncomfortable to sit and lay down, and I find that it does affect my life because I can’t go out and do everything that I want to do or see people because, you know, my legs are too big and my feet are too big and I can’t walk around and then I’m—and I’m uncomfortable sitting down and it makes, uh, social settings increasingly awkward.” (31-year-old, female, MELD-Na score of 16, primary sclerosing cholangitis)
	Need for support	“As for activity, I can’t barely do anything right now. It’s just too much for me. My boyfriend does most of the stuff for me right now, so I’m sick.” (56-year-old, female, MELD-Na score of 18, viral hepatitis)
		“Yep, yoga, uh, and knee brace, I can’t afford that. I would say I always had good results with chiropractic and even for that matter from the bottom of my feet, in the balance, and so on and so forth, What are they called? The physiotherapist people? It’s like, I mean, I do my best to try to figure out how to improve my balance. But it’s not the easiest thing to motivate yourself on a day-to-day basis, and you’re there, or you’re gone. Without any money, I can’t do nothing.” (63-year-old, male, MELD-Na score of 9, alcohol-related liver disease)

### Theme 1: Physical experience with pain

The physical experience with pain was common for all participants. Visceral pain was described in the upper areas of the abdomen, lower extremities, back, and shoulders. Sensory experiences encompassed the duration and intensity of pain, often described as constant or persistent, with some participants reporting pain lasting up to 4 years. Pain intensity varied by anatomical site, with the presence of fluid accumulation and swelling in the abdomen and lower extremities often associated with unrelieved pain. This visceral pain, frequently linked to ascites and edema, was described as intense and debilitating, contributing to overall physical discomfort. This is illustrated by the following quote:

The bloating in my stomach makes my back hurt ’cause it’s pressing the pressure, right. It makes it hurt real bad so. But I still try. I still get out and walk a little bit like walk around, loosen up. And then I gotta sit back down in my wheelchair because it’s just too much for my legs to handle. The pain everywhere is unbelievable. (56-year-old female participant diagnosed with viral hepatitis)

Musculoskeletal pain, reported by participants, included abdominal swelling that exacerbated preexisting conditions, such as arthritis, back pain, and umbilical hernia, leading to additional unrelieved pain. This pain was often compounded by symptoms manifesting across multiple regions, contributing to generalized discomfort. Those who had preexisting back injuries explained that the presence of abdominal swelling exacerbated this problem. Less commonly, participants reported paresthesia and headaches, with headaches likened to migraines and often unrelieved by medications like Tylenol. One participant noted itchiness from paracentesis needle insertions leading to painful tissue injury.

Functional limitations were significant with pain hindering participants’ ability to perform daily activities independently, such as attending school, grocery shopping, or leaving the house. Pain particularly restricted ambulation, reducing independence. Some participants avoided walking when pain intensity reached high levels.

If it’s like today, it’s not bad, but at 6 it’s fine. You know it’s when it’s like at 10 or whatever, like, say when it gets like really, really bad. I ain’t gonna be walking I’ll be here. I’ll be sitting in the backyard getting some sun and that’s it. (43-year-old female participant diagnosed with alcohol-related liver disease)

Pain disrupted household tasks, such as washing dishes, and slowed or halted activities. It also prevented engagement in paid employment, with employed participants noting they needed to wait for pain to subside before resuming work duties. These functional limitations highlight the pervasive impact of pain on physical independence and daily functioning, aligning with the biopsychosocial model’s emphasis on physical consequences of pain.^21^

### Theme 2: Psychological experience with pain

The psychological impact of pain was experienced by all participants. Fatigue, characterized as pervasive tiredness, drained participants’ energy, hindering their ability to manage pain’s emotional and physical demands. Participants noted that pain-induced fatigue exacerbated emotional distress, creating a cyclical effect.

And the fatigue that comes with pain. I think there’s a big correlation between being really tired and pain. It wears you out, it wears you out [crying]. (49-year-old female participant diagnosed with alcohol-related liver disease)

Emotional distress was prevalent, with pain impairing self-care and contributing to frustration and low mood. One participant noted pain exacerbated their depression, amplifying psychological strain. Coping strategies included pharmacological and nonpharmacological approaches, but participants often faced limited or ineffective pain management options. Participants identified Tylenol and hydromorphone as pharmacological treatments frequently prescribed to them for pain. However, they described Tylenol as not being able to fully relieve pain and hydromorphone as having serious side effects such as nausea and emesis.

Oh well, Extra Strength Tylenol can bring [the pain] down to like a 5 [out of 10]. But, I mean, it’s it doesn’t necessarily sustain it. But it does offer relief, and quite often I don’t take Tylenol all that often, but right. Uh, what do I do? (21-year-old female participant diagnosed with primary sclerosing cholangitis)

As for nonpharmacological interventions, participants reported using mindfulness, meditation, lying in bed, and listening to music. They described how these interventions distracted them from their frustration and offered some semblance of relief.

So every few minutes I would lay down. When I get up again and take my walk in the house or whatever exercise I do in the house. Until such time it’s been painful again. I may just lay down because of my pain. Because it’s a relief to me if I lay down. (64-year-old female participant diagnosed with viral hepatitis)

Acceptance was employed by the participants as a coping strategy to manage pain. Uncontrollable pain shaped their expectations and limited their engagement in daily and social activities, further impacting emotional well-being. Participants expressed frustration with health care providers’ inability to clarify pain causes or offer effective pain management, contributing to emotional distress. Changes in self-concept were noted, with participants concerned about others’ perception of their reduced physical abilities, conflicting with their desired self-image.

I have to accept that, uh, for a person that was the rock of the family to actually concede that you can’t do it all anymore. It it’s frustrating [crying]. (67-year-old male participant diagnosed with metabolic dysfunction–associated steatotic liver disease (MASLD)

Pain interference with mood, sleep, and enjoyment of life was significant, with pain disrupting social interactions and causing participants to withdraw from their social network, exacerbating emotional distress.

Uh, very much, yeah. But like I mentioned before, I have pretty much ostracized myself. (51-year-old male participant diagnosed with alcohol-related liver disease)

Pain significantly disrupted sleep, with participants reporting that pain prevented quality rest, further compounding emotional and physical challenges.

Yes, I am waking up during the evening. I have during the night. It’s the pain. The pain, it causes me to wake up. You just can’t sleep when it hurts. (53-year-old female participant diagnosed with unknown cause of cirrhosis)

Sleep impairment limited participants’ social engagement and enjoyment of life, exacerbating their ability to manage pain’s physical and emotional demands. These psychological impacts highlight the interplay between pain and emotional well-being, reinforcing the need for multidimensional pain management strategies.

### Theme 3: Sociocultural experience with pain

As noted above, pain impacted social experiences and was dependent on the unique cultural expectations of each study participant. Social disruption was evident, with pain interfering with participants’ ability to maintain reciprocal relationships, limiting social engagement. Participants described a strong need for connectedness and social support. However, they were less able to sustain a reciprocal or balanced exchange of support with others due to their pain, fatigue, and decreased mobility. Many expressed feeling dependent on intimate partners for their physical care, food, and activities of daily living. The participants increased their reliance for support when their pain increased.

Yeah, I think, um, for the most parts, um, it doesn’t affect us too much. My significant other, you know, is aware when I do have pain is just more, um, you know, conscientious. Uh, you know, gives me more support at those times. (48-year-old male participant diagnosed with primary sclerosing cholangitis)

Most participants reported a need for support due to their inability to engage paid employment. Financial concerns conflicted with social and/or familial expectations to be independent and support others. One participant indicated that their monthly disability funding was not sufficient to cover their expenses, thereby leading to recurrent financial crises. Another participant expressed concerns with their inability to afford nonpharmacological pain treatments such as chiropractor, yoga classes, or even a knee brace.

Finally, several participants described lower proactive access to health services during the COVID-19 pandemic. In addition, they socialized with family and friends less often, which further intensified their need for support during episodes of pain exacerbation. This increased reliance on support systems highlights the sociocultural burden of pain, where social isolation and financial strain compound the challenges of living with cirrhosis.

## Discussion

This qualitative descriptive study explored the multidimensional experience of pain in adults with cirrhosis, highlighting its profound impact across physical, psychological, and sociocultural domains.^13,21^ Persistent, unrelieved pain significantly impaired participants’ physical function, emotional well-being, and social interactions.^13,21^ Physical pain contributed to impairment and exacerbated old injuries.^3,11^ Pain contributed to fatigue and decreased the ability to mobilize both inside and outside the home.^13,21^ In turn, pain interfered with the ability to live independently.^3,13^ Participants were dependent on others to assist with housework, food preparation, and transport.^13,18^ The inability to sustain paid employment was a common challenge. Reciprocal relationships with others were significantly constrained.^33^ Limited pain treatment options hindered participants’ abilities to concentrate on aspects of life beyond their pain, effectively diverting their attention solely toward managing their pain.^13^ Personal self-concept was negatively impacted, and participants described a misalignment between social expectations and whom they aspired to be.^31,33^

Our study revealed that persistent, unrelieved pain was a common experience among participants, aligning with the broader literature on pain’s adverse effects on physical health.^13,51,52^ Pain carries significant consequences; for example, Cortesi et al.’s^53^ longitudinal, observational study involving a sample size of 2962 patients with chronic liver disease found that patients with cirrhosis were more likely to have mobility issues when compared to the general population. Such mobility limitations can lead to decreased physical activity. Considering that the participants in our study reported increased fatigue, exercise intolerance may manifest.^54,55^ Given the burden of pain and fatigue in this population, clinical recommendations should emphasize comprehensive pain management strategies tailored to the person’s needs. For instance, integrating physical therapy, structured exercise programs, and nonhepatotoxic analgesics into hepatology clinics can mitigate pain and improve functional capacity. Patient education on pacing activities, optimizing nutrition, and exploring mindfulness-based interventions can further support muscle function and emotional well-being. Further research is needed to explore targeted interventions that address both pain and fatigue while considering unique challenges faced by each individual with liver disease. The dynamic interplay of physical, psychological, and sociocultural domains, as highlighted by the biopsychosocial model, signifies the need for holistic interventions that address these interconnected challenges.^56^

Unrelieved pain poses a significant challenge for patients with cirrhosis and their clinicians. The primary remedy for pain management reported by the participants continues to be pharmacological intervention. For example, the participants in this study commonly mentioned resorting to Tylenol to alleviate pain. However, analgesia alone was often ineffective for pain relief, leading some to turn to opioid analgesics such as hydromorphone to more effectively address their pain. Unfortunately, the emergence of adverse side effects such as nausea and vomiting served as a deterrent for some to treat their pain. The trade-off between adverse side effects and pain relief may explain why patients inconsistently use prescribed opioids. Rogal et al.’s^56^ retrospective cohort study that analyzed 1286 patient charts to examine factors associated with pain and opioid use in patients with chronic liver disease and found higher pain ratings among patients with prescribed opioids. This may suggest that despite opioids being intended for pain relief, the negative side effects might deter regular use. Given the limitations of pharmacological approaches, some participants turned to self-management strategies with minimal guidance. Rest was a commonly reported strategy, though its timing and duration varied among individuals. Hansen et al.’s^15^ prospective longitudinal descriptive study of 20 patients with end-stage liver disease found significant variability in self-management practices for pain relief. This inconsistency can lead participants to explore alternative approaches that they may deem plausible. One promising nonpharmacological intervention is mindfulness-based strategies. Bajaj and Pande’s^57^ study on mindfulness and resilience suggests that mindfulness plays a key role in improving well-being by fostering resilience, reducing stress, and enhancing emotional regulation. Mindfulness-based interventions, such as structured mindfulness-based stress reduction programs, can be integrated into hepatology care to provide low-risk, evidence-based options for managing pain and emotional distress, particularly for patients with limited pharmacological options due to liver dysfunction.^58^ Future research should explore structured mindfulness interventions tailored to individuals based on different liver disease severity particularly addressing pain, fatigue, and emotional distress.

The intersection of pain and the need for support for individuals with cirrhosis represents a challenging and complex aspect of pain management. Participants in this study described physical, psychological, and social limitations resulting from chronic pain, which often impeded their ability to engage in daily activities and employment. Beyond the physiological burden of pain, the emotional toll of living with liver disease can be profound. Social support plays a critical role in mitigating these challenges, yet many participants expressed a lack of accessible support systems to help them navigate their condition. Existing research highlights the importance of strong social networks to improve health-related quality of life, enhance coping mechanisms, and reduce the psychological distress associated with chronic conditions.^59^ Though financial strain was not a primary theme in this study, it is important to acknowledge its impact on patients with liver disease. Ufere et al.’s^60^ scoping review on the financial burden of chronic liver disease highlights the significant economic strain experienced by patients and their caregivers, with associations between financial distress, increased health care utilization, and poorer health outcomes. Participants in our study expressed the lack of financial resources to obtain necessities such as food, clothing, and other essentials. The impact of pain on physical, psychological, and social abilities of the participants can impede their capacity to engage in paid employment,^61^ resulting in a cycle of financial distress. For instance, cognitive behavioral therapy, acupuncture, and transcutaneous nerve stimulation have been identified as possible nonpharmacological interventions to pain in the most recent practice guidance.^14^ However, most of these therapies comprise out-of-pocket fees that require the person to have the financial resources to receive the service. Webster et al.^61^ used the term “chronic struggle” as a concept to describe the vicious cycle these participants engage in. They need resources to treat the pain but are unable to receive those resources because their pain prevents them from obtaining the financial resources to pay for the pain treatment. The financial strain associated with pain is not only limited to the tangible costs but also impacts mental health. The stress and anxiety stemming from economic uncertainty can exacerbate the experience of pain, thereby creating a cycle that further diminishes the quality of life of the person.^13,19,21,62,63^ Addressing the intricate relationship between pain and the socioeconomic needs of a person requires a comprehensive approach to multidimensionality that explores the needs of the person beyond the conventional medical interventions of pain management.

## Strengths and limitations

This is the first Canadian qualitative study focused on the multidimensionality of pain experiences for persons with cirrhosis. Our study highlights the need for future intervention studies to address the unrelieved pain experienced by this population impacting every aspect of their daily lives. Furthermore, our use of a conceptual framework and an interprofessional team of researchers strengthens our results.

One limitation of this study is that ethnicity was not explicitly addressed, which may influence pain experience. There is evidence to suggest that ethnicity can significantly impact pain perception and management. For instance, Green et al.^64^ found that African Americans and Hispanics were more likely to experience severe pain and receive less adequate pain management compared to their non-Hispanic patients. This disparity is often attributed to systemic biases, cultural differences in pain expression, and varying levels of access to health care.^65^ Future studies should aim to include diverse populations and examine how cultural, social, and systemic factors contribute to pain experiences. Additionally, researchers should explore interventions that address these disparities, such as culturally competent care, patient education, and advocacy, to ensure equitable pain management across all racial and ethnic groups. By doing so, we can improve pain outcomes and enhance the quality of life for all patients with cirrhosis.

Though the biopsychosocial model of pain^21^ provides a robust framework for understanding the multidimensional nature of pain, it may not be entirely sufficient for a holistic approach to pain in patients with cirrhosis, particularly given its lack of attention to spirituality. Spirituality can play a significant role in how patients perceive and manage pain, especially in chronic and life-limiting conditions.^66^ The integration of spiritual services can provide a more holistic understanding of the patient’s pain experience. Future research should explore the relationship between pain and spirituality, emphasizing how spiritual care interventions can be effectively incorporated into pain management strategies for patients with cirrhosis, ensuring a truly multidimensional approach to care.

Another limitation of this study stems from the convenience sampling method employed, leading to unequal representation of participants based on severity of liver disease. This suggests that our findings may be more reflective of voices from participants with lower severity of liver disease compared to those with more severe liver disease. Recruitment during the COVID-19 pandemic may have limited the range of patients available in clinic for recruitment. However, it is important to note that this study is one of the first exploring the impact of pain on a multitude of domains.

## Conclusion

Pain is a multidimensional experience that encompasses a wide range of challenges faced by persons with cirrhosis. Study participants described the negative impact of pain on various dimensions of their lives, including physical function, emotional well-being, and social interactions. They experienced a range of challenges due to persistent, unrelieved pain, including need for support, compounding symptoms, and limited treatment options available impacting mental well-being. Our findings underscore the persistent issue of unrelieved pain amongst the participants, emphasizing the need for further research to address the gaps in current pain management strategies.

## Supplementary Material

7 Supplemental App A Interview questions.docx

8 Supp App B COREQ.pdf

## Data Availability

All available data in this qualitative study are referenced throughout the paper as Figures 1 and 2, Tables 1 and 2, and Supplemental Appendices A and B.
