# Supplementary material for: Multidimensional experience of pain in adults with cirrhosis: A qualitative descriptive study
Source: Can J Pain. 2025 Dec 15;9(1):2573273. doi: 10.1080/24740527.2025.2573273 (PMC12707517; doi:10.1080/24740527.2025.2573273)
Supplement: 7 Supplemental App A Interview questions.docx [file UCJP_A_2573273_SM2182.docx]

## Supplemental Appendix A. Semi-structured interview guide

| Interview question item # | Questions |
| --- | --- |
| 1 | Do you experience pain in your body? |
| 2 | What medications do you take when you experience pain? |
| 3 | What other treatments or activities do you use for pain? For example, taking a nap, reducing activity, taking a bath, listening to music, or talking to someone.  How much relief have these other treatments or activities for pain provided? For example, 0% (No relief) 10% 20% 30% 40% 50% 60% 70% 80% 90% 100% (Complete relief) |
| 4 | How has pain affected your General Activity?  For example, how has pain affected your ability to go to the grocery store, drive, or take public transportation? |
| 5 | How has pain affected your Walking ability?  For example, how has pain affected your ability to walk around your home, neighborhood or even to the store? |
| 6 | How has pain affected your Normal Work (includes both work outside the home and housework)?  For example, how has pain affected your ability to go to work, cook, or to do laundry? |
| 7 | How has pain affected your Sleep?  For example, does the pain affected your ability to fall asleep or stay asleep at night? |
| 8 | How has pain affected your Mood?  For example, how has pain affected your ability to feel happy, optimistic, or hopeful? |
| 9 | How has pain affected your Relations with Other People?  For example, how has pain affected your relationship with your family, friends, significant others, or co-workers? |
| 10 | How has pain affected your Enjoyment of Life?  For example, how has pain affected your ability to enjoy life such as doing the things you want to do, when you want to do them? |
| 11 | Has the COVID-19 pandemic changed how you manage your pain? If yes, how so?” |
| 12 | How does your finances affect your experience with pain?  For example, do you have enough money to pay for treatments? |
